# Milk or Kefir, in Comparison to Water, Do Not Enhance Running Time-Trial Performance in Endurance Master Athletes

**DOI:** 10.3390/nu16050717

**Published:** 2024-03-01

**Authors:** Kristen N. Gross, Patrick S. Harty, Joesi M. Krieger, Petey W. Mumford, Kyle L. Sunderland, Anthony M. Hagele, Chad M. Kerksick

**Affiliations:** Exercise and Performance Nutrition Laboratory, College of Science, Technology and Health, Lindenwood University, St. Charles, MO 63301, USA; pharty@lindenwood.edu (P.S.H.); jkrieger@lindenwood.edu (J.M.K.); pmumford@lindenwood.edu (P.W.M.); ksunderland@lindenwood.edu (K.L.S.); ahagele@lindenwood.edu (A.M.H.)

**Keywords:** kefir, probiotics, athletes, recovery

## Abstract

This study compared flavored kefir (KFR) and flavored milk (MLK) as a recovery drink in endurance master athletes. Using a randomized, placebo-controlled, non-blinded crossover design, 11 males and females completed three testing visits whilst acutely ingesting either KFR, MLK, or water as a placebo (PLA). KFR supplementation occurred for 14 days before the KFR-testing day, followed by a 3-week washout period. Testing visits consisted of an exhausting-exercise (EE) bout, a 4-h rest period where additional carbohydrate feeding was provided, and a treadmill 5 km time trial (TT). The Gastrointestinal Symptom Rating Scale (GSRS) survey was assessed at four timepoints. Blood was collected at baseline and after the TT and was analyzed for I-FABP levels. No significant difference (PLA: 33:39.1 ± 6:29.0 min, KFR: 33:41.1 ± 5:44.4 min, and MLK: 33:36.2 ± 6:40.5 min, *p* = 0.99) was found between the groups in TT performance. The KFR GSRS total score was significantly lower than the PLA after EE (*p* = 0.005). No differences in I-FABP were observed between conditions. In conclusion, acute KFR supplementation did not impact TT performance or I-FABP levels but may have reduced subjective GI symptoms surrounding exercise when compared to MLK or PLA.

## 1. Introduction

Following long and/or intense exercise, a recovery period is important to allow athletes to repetitively train with high volumes at challenging intensities. Ideal recovery centers upon the delivery of appropriate amounts of macronutrients balanced with adequate fluid and energy consumption alongside restorative amounts of rest. Many situations exist in sports competitions or training where athletes have short (<6 h) recovery windows (i.e., participation in a tournament or training camp). To facilitate timely recovery, nutritional recommendations advocate for aggressively consuming carbohydrates (CHO) immediately upon completion at a rate of 1.0–1.2 g/kg/hour to promote maximal glycogen resynthesis [1]. Although the focus in recovery should be on CHO intake, the addition of protein has been found to further augment glycogen resynthesis rates, especially in situations where the intake of CHO was lower than the recommended amount in the first four hours of recovery [1,2,3,4]. The suggested amounts of co-ingestion of CHO and protein to maximize glycogen storage are 0.8 g/kg/h of CHO and 0.4 g/kg/h of protein [2,3,5]. Bovine milk with chocolate or strawberry flavoring (MLK) provides favorable amounts of CHO, protein, vitamins, and minerals while also serving as an efficient means to replace lost fluid from exercise [2,6]. For these reasons, MLK has become a popular and efficacious option as a recovery beverage for athletes [2,6].

In addition to creating nutritional challenges, intense exercise and endurance training can also temporarily increase inflammation and compromise the immune and digestive systems in athletes [7,8,9,10]. High-intensity or prolonged exercise can temporarily suppress natural killer (NK) cells, leaving athletes more susceptible to illness in the first few hours after an exercise session or competition [7,11,12,13,14]. Additionally, gastrointestinal (GI) symptoms, such as nausea, bloating, diarrhea, cramps, and pain, often develop in connection with prolonged exercise, particularly in running, and have been documented to occur in up to 50% of endurance runners [8,15,16,17]. The etiology of these GI issues is thought to begin with reduced blood flow to the GI tract during exercise, causing increased permeability of the luminal membrane, which can result in systemic endotoxemia and, ultimately, GI symptoms [8,10,18,19,20]. Damage to the endothelial cells can initiate an acute inflammatory response with the release of cytokines, such as IL-6, which usually induces additional cell signaling resulting in a heightened inflammatory response [9,17]. Jeukendrup et al. [15] found that the increase in IL-6 correlated with GI symptoms and reported a 27-fold increase in IL-6 immediately after an Ironman distance triathlon race. Therefore, IL-6, which is produced in and released from skeletal muscle, is often used as a blood-based biomarker to track the physiological changes that correlate with inflammation and GI symptoms in conjunction with exercise and nutritional interventions [15,17,19,21,22]. Likewise, I-FABP is a protein found in the intestinal mucosa involved in the transportation of fatty acids. During prolonged exercise sessions, the reduction of blood flow can cause I-FABP to be found at increased levels in the blood, thus making it a valuable blood-based biomarker for enterocyte damage that increases with exercise [15].

To combat the increased risk of contracting mild illnesses or experiencing GI symptoms and heightened inflammation after challenging exercise, athletes are increasingly exploring the use of probiotics [7,8,23]. Probiotics are defined as “live organisms that when administered in adequate amounts confer a health benefit on the host,” [24], and have been shown to support the immune and digestive systems when regularly consumed. Probiotics are being utilized for their ability to modulate immune function resulting in many benefits for athletes including reductions in both inflammation and GI symptoms [7,8]. A meta-analysis has reported probiotics to be effective in attenuating the increase of IL-6 in endurance runners, cyclists, and triathletes supplementing for 4–11 weeks with species ranging from *Lactobacillus plantarum* PS128, *L. acidophilus*, *Bifidobacterium bifidum*, *B. animalis* subsp. *lactis*, and/or *L. fermentum* with CFUs (colony forming units) ranging from 25 × 10^9^–1.5 × 10^10^ [21]. Additionally, Mooren et al. [17] showed that daily supplementation with a 5 mL ampulla containing the probiotic *Escherichia coli* strain Nissle 1917 (Mutaflor) with approximately 1 × 10^10^ CFUs for 28 days in untrained men resulted in a significant reduction in I-FABP levels after exhaustive treadmill exercise compared to the placebo group [17,19,22]. 

Kefir is a fermented milk made from kefiran grains that contains probiotic bacterial and yeast cultures, and, when it is a flavored variety, contains similar amounts of CHO and protein to MLK. For these reasons, flavored kefir (KFR) may exhibit similar properties as a recovery drink while also supporting the immune and digestive systems in response to challenging exercise. However, limited research in exercising humans has been conducted using kefir. For example, O’Brien et al. [25] randomly assigned 67 males and females in a parallel fashion to one of four groups to examine outcomes associated with exercise performance: (1) placebo + normal physical activity, (2) kefir + normal physical activity, (3) placebo + endurance exercise training, and (4) kefir + endurance exercise training. Each dose was consumed two times each week and delivered 10^9^–10^10^ CFUs per dose over the 15-week supplementation protocol. Performance was assessed using a 1.5 m walk/run [25]. Compared to the two normal physical activity groups, significant improvements in exercise performance were found in the two exercise groups, but no significant differences were identified between the exercise training + kefir and exercise training + placebo groups [25]. However, lowered C-reactive protein (a marker of inflammation) levels were found only in the exercise + kefir group compared to the baseline measurements in the same group [25]. A crossover trial (with a 3-week washout) was completed by Lee et al. (2021), whereby 16 human males were supplemented for 28 days with sachets containing 20 g of lyophilized cultures isolated from SYNKEFIR made with a starter culture containing *L. paracasei* DSM 32785 (LPC12), *L. rhamnosus* DSM 32786 (LRH10), *L. helveticus* DSM 32787 (LH43), *L. fermentum* DSM 32784 (LF26), and *S. thermophilus* DSM 32788 (ST30), or a placebo [26]. A test-to-exhaustion protocol indicated statistically significant gains in exercise performance in the kefir cultures group when compared to its performance prior to supplementation, as well as significantly lower lactate levels. Changes in gut microbiota diversity were not observed [26]. Another investigation by Wang et al. [27] examined the ability of kefir to alleviate GI symptoms. This study randomly assigned 54 males and females to two supplementation groups (placebo or kefir) and had them supplement daily for three weeks while evaluating changes in GI symptoms. A significant reduction in abdominal bloating in the kefir group was reported [27].

In summary, adequate nutrition is a critical factor in optimizing recovery from intense, stressful, and depleting exercise. The intake of efficacious amounts of CHO and protein during the early hours of recovery is a well-established strategy to positively impact many aspects of recovery. While probiotics have been shown to support immune and digestive health, more research is needed to understand if adding probiotics to a CHO + protein combination can further augment exercise recovery and impact subsequent exercise performance, as well as affect GI symptoms, biomarkers of inflammation and enterocyte damage. The primary aim of this study was to determine if the addition of probiotic cultures to a CHO and protein recovery beverage resulted in benefits to subsequent exercise performance. A secondary aim was to determine if the addition of probiotics to the CHO and protein beverage impacted subjective ratings of GI symptoms and serum concentrations of IL-6 (a marker of inflammation) and I-FABP (a marker of enterocyte damage).

## 2. Materials and Methods

### 2.1. Overview of Research Design

**Figure 1:**
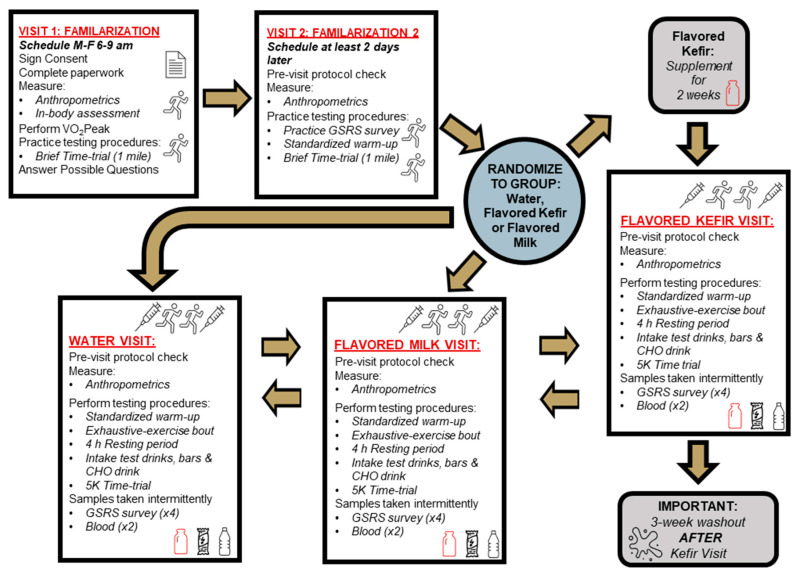
Flowchart of study visits. h = hours, CHO = carbohydrate, K = kilometer, GSRS = Gastrointestinal Symptoms Rating Scale. Red underlined text indicates study visits. Bold text indicates importance of instructions.

In a randomized, crossover fashion, eleven healthy male and female master endurance athletes completed three total testing visits for three non-blinded conditions: PLA, KFR, and MLK (Figure 1). To avoid the need to prematurely open containers to accurately measure KFR and MLK and create a potential risk of contamination and other considerations, the decision was made a priori to not blind the administration of the supplements to the participants or the research staff. This decision was made for a few reasons. First, to deliver doses that were as close to being isocaloric and equivalent in terms of carbohydrate and protein as possible, slightly higher kefir amounts needed to be administered, resulting in slightly different volume amounts between conditions. Moreover, this step would have required the utilization of a 3rd party container that would not have been commercially sterilized and pasteurized whereby the assigned volume of each condition may not remain fresh for consumption during the duration of each testing condition. Lastly, KFR knowingly has a different texture and flavor profile, which would have been close to impossible to match without further changing the nutrient profile. As such, we decided to conduct an open-label study to avoid these challenges and to promote the safe interactions of our participants with the study protocol.

Approval for this human research study was obtained from Lindenwood University Institutional Review Board (IRB-23-71; approval date 23 January 2023) with all ethical considerations aligning with the Declaration of Helsinki. This trial was retrospectively registered at ClinicalTrials.gov (accessed on 16 November 2023) with the identifier NCT06165523. Participants were invited to the laboratory to complete screening and their first familiarization visit, during which the study protocol and design were explained, and informed consent was obtained. After obtaining consent, anthropometrics and body composition measurements were completed for exclusionary and descriptive purposes. Following a standardized warm-up, eligible participants had their VO_2_Peak assessed. Then, after observing a brief (15–20 min) break during which food logs and additional protocols were explained, participants practiced a one-mile time trial to foster acclimation to the non-motorized treadmill. Visit 2, scheduled at least two days after visit 1, was another one-mile time trial familiarization of the non-motorized treadmill, as well as of the GSRS survey.

The three exercise testing days (visits 3–5) followed the same protocol as Karp et al. [6]. Visit 3 occurred at least two days after visit 2, and each subsequent study visit was separated by at least one week. Visits 3–5 consisted of a pre-visit compliance check, a standardized warm-up, an exhaustive-exercise (EE) bout with the intent to deplete glycogen, then a four-hour recovery period (RP) that included recovery supplementation of the assigned treatment beverage, and further glycogen replenishment dispersed at consistent times throughout the RP, followed immediately by a 5 km (5K) time trial to test the impact of the assigned supplementation on exercise performance. The EE and 5K exercise bouts were both performed in order to simulate the repeated exercise situation that athletes are sometimes required to perform, allowing for the testing of the recovery beverages that would then be used in this setting. Prior to each study visit, participants were provided information to standardize their diet to include a high CHO intake the day before testing, fast the night before testing, and finally with completion of the EE. Due to the need to allow for colonization of the microbiota communities found within KFR, a two-week supplementation of KFR was carried out [8,28,29] which ended on the subsequent testing day to evaluate the efficacy of KFR followed by a washout period of approximately 3 weeks. The pre-supplementation was deemed not necessary for the MLK or PLA conditions since neither beverage contained probiotic strains. During the testing visits, the participants also had their height, weight, blood pressure, and heart rate measured. Serum blood samples were collected at baseline and after the 5K to evaluate changes in IL-6 and I-FABP. The GSRS was assessed at baseline, post-EE, post-RP, and post-5K time trial. The EE distance, total time of EE, total water intake (mL) during the EE, 5K performance times, and ratings of perceived exertion (RPE) during the time trial were also all recorded for each testing visit. Exercise sessions for all participants started between 0600–0830 h, with each participant starting each subsequent testing visit at a similar time as the first visit, and data collection was completed under the supervision and direction of trained graduate students and faculty at the Exercise and Performance Nutrition Laboratory and the Athlete Training Center at Lindenwood University.

### 2.2. Participants

Due to the exploratory nature of this study and the rigorous protocol, the target sample size for this study was 10–15 participants. Eleven healthy females and males between 40–75 years of age who were free from disease, as determined by completion and review of a medical history, were eligible for this study. The participants were required to be well-trained and to have trained aerobically at least three times per week for a weekly total of at least 180 min. ‘Well-trained’ in this study was defined as those individuals that met two of the four following criteria: aerobic training frequency of 3–7 sessions per week; duration of training sessions at least 60 min; at least three years of consistent aerobic training; or raced at least once per year [30].

**Figure 2:**
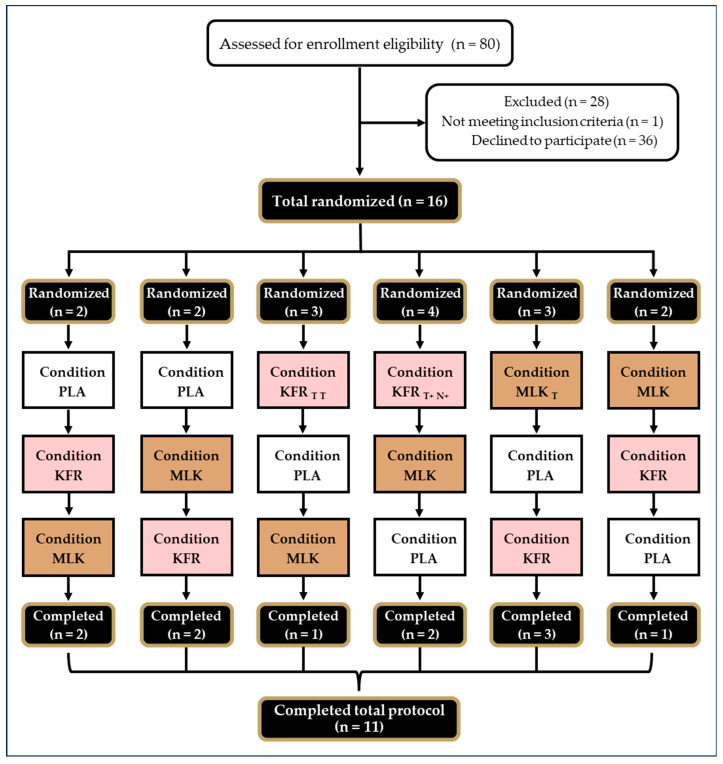
CONSORT (Consolidated Standards of Reporting Trials) Diagram. Clear boxes with rounded edges represent the screening stage of the trial. Black boxes with gold lines indicate the number of participants that were randomized or that completed the trial. Clear, pink or brown boxes convey the condition randomization order: clear = PLA, pink = KFR, brown = MLK. T = participant dropped due to lack of time, T+ = participant dropped due to lack of time after KFR pre-supplementation, N+ = participant dropped due to injury during EE after KFR pre-supplementation.

Additionally, any individual who had ever been diagnosed with or was currently being treated for any cardiac, musculoskeletal, pulmonary, immunological, or metabolic disease, or had been treated for cancer within 5 years, was excluded from this study. Individuals with a body mass index (BMI) greater than 30 kg/m^2^, or a body fat percent greater than 25% fat for males and 30% fat for females, were excluded. Individuals who were lactose intolerant or had an allergy to the study products were excluded. Any individual who was currently, or recently had been, taking probiotics or antibiotics and was unwilling to do a washout period (4 weeks for probiotics; 5 weeks for antibiotics) was excluded from this study. Any individual who was currently supplementing with biotin and was unwilling to refrain from taking it, at a minimum of 72 h before visits 3–5, was excluded due to the potential interference with the analysis of biomarkers. Additionally, individuals who were unwilling to follow pre-visit or supplement protocols were excluded. CONSORT information is reported in Figure 2.

### 2.3. Dietary and Physical Controls

Between the first familiarization and first testing visits, participants completed a 3-day food log using the app MyFitnessPal^TM^ (v23.5.0) to record all food and calorie-containing fluids consumed. Each log was analyzed for total kilocalories and macronutrients consumed. To obtain similar relative glycogen storage levels between all the participants, they were instructed to follow a high-CHO diet (8 g of CHO/kg body weight) to ensure adequate muscle glycogen content before their first testing visit. When pre-supplementing with KFR, participants were instructed to adjust their repeated high-CHO diet accordingly to allow for the addition of the macronutrients of the two servings of KFR. Each participant’s carbohydrate intake goal was calculated, provided in grams, and given to each participant during the first familiarization visit along with a list of high-CHO foods and suggestions to help obtain their daily carbohydrate target. Participants then logged their high-CHO foods asDay 3 into MyFitnessPal^TM^ which allowed them to track the amount of CHO they had ingested so far that day. Participants were then asked to replicate their high-CHO diet the day before each of their additional two testing visits. Participants were required to not take antibiotics or consume any other probiotics, including yogurt or other fermented foods, and to remain consistent with their normal dietary intake of prebiotics and general diet during the length of the entire study [31]. During the 2-week pre-supplementation period, all participants were required to fill out a log tracking their supplementation regimen.

All participants were required to refrain from strenuous exercise for 72 h prior to each study visit and any type of exercise for 24 h before study visits to eliminate confounding fatigue prior to completing the study protocol. Participants were asked to arrive in a fasted, well-hydrated state, wearing the same running shoes and similar clothing for each of their visits. Additionally, participants were asked to keep their training consistent throughout the entirety of the study and to refrain from caffeine, nicotine, and alcohol intake for 12 h before visits.

### 2.4. Interventions

The most consistently available flavors from a local shopping market (chocolate milk and strawberry kefir) were used for the intervention for convenience and the real-world application of this approach. Due to slight deviations in the macronutrient content, a 240 mL serving of the strawberry-flavored low-fat kefir used in this study (Lifeway Foods, Inc. Morton Grove, IL, USA) contained 18 g of CHO, 10 g of protein, 2 g of fat, and 130 calories, while a 240 mL serving of the low-fat chocolate milk used in this study (Schnucks, St. Louis, MO, USA) contained 24 g of CHO, 8 g of protein, 2.5 g of fat, and 150.5 Kcal. Water was used as the PLA.

### 2.5. Supplementation Protocol

At the end of the second familiarization visit, the participants were randomized into their first condition group using random allocation software Research Randomizer; www.randomizer.org (accessed on 13 February 2023). When assigned to the KFR group, the participants consumed their first dosage while at the laboratory upon completion of their current visit, then were given enough KFR to supplement for two subsequent weeks before returning to the laboratory for the acute exercise testing of their assigned recovery test beverage. Following Lifeway Foods’ recommendations to maximize gut modulation, participants were asked to consume 240 mL of KFR twice per day for 14 days, a total of 28 doses. Supplementation logs were given to the participants to track whether both doses were taken on the required days. The participants were instructed to consume each dose immediately after exercising, or 0–30 min prior to breakfast if not exercising that day, and 0–30 min before dinner for the second dose [31]. To prevent adhesion to the sides of the kefir container, participants were told to shake the container before pouring 240 mL into a certified measuring container that contained measurement indicators on the side of the container. To prevent any loss of product, participants were told to add ~100 mL of water to the container after their initial ingestion of the kefir and agitate it before consuming all of the remaining water–kefir liquid. Before moving on to any incomplete testing visits, a three-week washout period occurred after the KFR condition to allow for restoration of the gut microbiota.

### 2.6. Acute Supplementation Protocol

**Figure 3:**
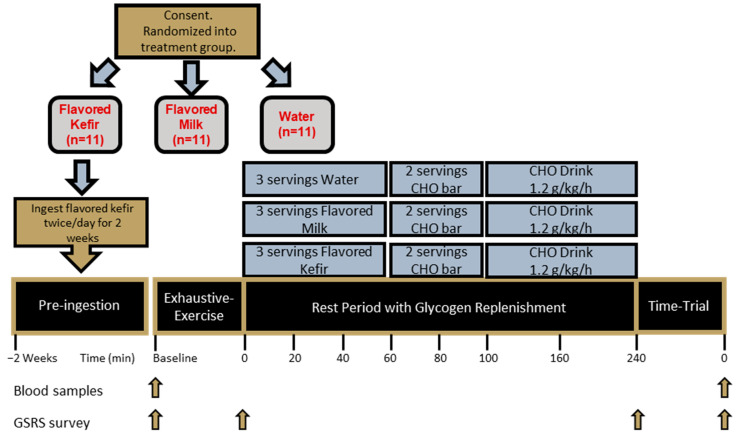
Schematic overview of glycogen replenishment for testing days. Gold box indicates that a participant consented and was randomized. Gray boxes with red text convey that randomization occurred into water (PLA), flavored milk (MLK), or flavored kefir (KFR). The gold box with an arrow conveys that a 2-week pre-supplementation will occur when the participant is randomized to this condition. The black boxes represent the testing day protocol, beginning with the exhaustive-exercise, the 4 h (hour) recovery period that includes replenishment shown in light blue boxes, and the 5K (kilometer) running time-trial performance test. Blood samples were taken when indicated with gold arrows, processed and analyzed for IL-6 (interleukin-6) and I-FABP (intestinal fatty-acid binding-protein) levels. The GSRS (gastrointestinal symptom rating scale) survey was also taken at timepoints designated by the gold arrows.

As part of the acute recovery period we employed and patterned a protocol based on Karp et al. [6], each participant consumed one serving, modeled from O’Brien et al. [25], of either PLA, KFR, or MLK immediately upon cessation of the EE, and additional servings 20 and 40 min after completion of the EE, to deliver equal amounts of CHO for the KFR or MLK conditions and no CHO or protein for the PLA (Figure 3). With the matched carbohydrate amounts, the total of the three servings (0, 20, and 40 min post-EE) was 72 g of CHO for KFR and MLK, 40 g of protein for KFR, 24 g of protein for MLK, 519 Kcal for KFR, and 451.5 Kcal for MLK. When randomized to PLA, participants consumed only water for the first 40 min post-EE immediately upon cessation of the EE and another 240 mL serving at both the 20 and 40 min marks post-EE. All beverage conditions were measured in a food-grade beaker and poured into a plastic disposable cup and were followed-up by an additional 100 mL of water to rinse out both the beaker and the serving cup (or for equal fluid amounts in the case of water). No additional fluids were given to the participants for the first 60 min of recovery.

In addition to the treatment drinks, additional recovery food and beverage drinks were provided to all participants, according to the methods of Dahl et al. (2019), to provide the recommended 1.2 g carbohydrate/kg of CHO/protein for four hours [4]. Two servings of granola bars (Nature Valley Crunchy Oats ‘N Honey) were used as the recovery meal, given at the 60 and 80 min post-EE marks, providing a total of 58 g carbohydrates, 6 g protein, and 14 g of fat for a total of 380 calories. A CHO (maltodextrin) recovery drink (Now Sports Nutrition Carbo Gain) was provided, containing 60 g of CHO per serving, and was flavored with fruit punch Crystal Light containing 2 g of CHO. It was distributed at a rate consistent with 1.2 g of carbohydrate/kg of body weight and consumed at the 100 min and 160 min post-EE marks. Participants also drank water ad libitum for all three exercise testing visits for the last three hours of the recovery period.

### 2.7. Anthropometrics

During the screening visit, the height of the participants was measured to the nearest ± 0.5 cm using an analog wall-mounted stadiometer (HR-200, Tanita Corp, Inc., Tokyo, Japan) with their shoes removed and standing erect on flat feet. A bioelectrical impedance analyzer was used at the first visit to determine fat and fat-free masses, and body fat percentage (InBody 570, Cerritos, CA, USA). Body composition analysis occurred between 0600 and 1000 h by trained research personnel according to device specifications, with participants removing jewelry and loose clothing articles and wiping their palms and foot soles with a wipe provided by the manufacturer (InBody tissue, InBody, Cerritos, CA, USA) before standing on the platform with hands and feet in contact with sensors.

At the start of all study visits, participants had their resting heart rate and blood pressure measured using an automated sphygmomanometer (Omron BP785, Omron Corporation, Kyoto, Japan) after being seated with their feet uncrossed on the floor for approximately five minutes. Body mass was measured using a self-calibrating digital scale (Tanita BWB-627A, Tokyo, Japan) and recorded to the nearest ±0.1 kg. Body masses recorded after visit 3 were compared to ensure participants were weight stable through the testing visits [32].

### 2.8. VO_2_Peak Assessment

At their first visit, participants completed a VO_2_Peak assessment by running on a Woodway Pro treadmill (Woodway USA, Inc., Waukesha, WI, USA) connected to a metabolic cart (True Max 2400 Metabolic Measurement System, ParvoMedics, Sandy, UT, USA) calibrated to within 2% of the previous day’s calibration value. A staged treadmill protocol with the grade held consistent at 1%, adapted from Jones et al. [33], was used, and began with a self-selected 5 min light jog. After the completion of the warm-up, the protocol began with three stages that were each three minutes long, followed by 2 min stages which gradually increased in speed until volitional exhaustion was reached [33,34]. Heart rate was continuously monitored throughout the test (Polar FT1, Polar, Kempele, Finland). It was considered a true VO_2_Peak assessment if three out of the following four criteria had been met: respiratory exchange ratio (RER) values met or exceeded 1.05; recorded heart rates were within ten beats of age-predicted maximal heart rate (Max HR = 208 − [0.7 × age]); there was a plateau of VO_2_ despite increasing workload; or the rating of perceived exertion (RPE) was 17 or greater. The participant’s VO_2_Peak was considered the highest attainable consumption of oxygen recorded in a 30-s interval. The participant’s VO_2_Peak was used to determine the different speeds during the exhaustive-exercise protocol.

### 2.9. Exhaustive-Exercise Protocol

Prior to the EE, participants completed a 5 min warm-up run on a motorized Woodway treadmill (Woodway DESMO-EVO, Woodway USA, Waukesha, WI, USA) at a speed corresponding to 40% VO_2_Peak. Participants then completed an exhaustive-exercise intermittent run protocol, modeled from Taylor et al. (2011), that was intended to deplete glycogen stores (EE). After a 1 min rolling start at 50% of their VO_2_Peak, the EE protocol commenced with a run set for two minutes at a treadmill speed calculated at 90% of the velocity at which their VO_2_Peak was achieved. This two-minute 90% VO_2_Peak period was followed immediately with a two-minute period with a speed calculated at 50% of their VO_2_Peak, for recovery. The 90%/50% VO_2_Peak work/recovery two-minute stages were repeated until the participant was no longer able to complete the stage at the speed of 90% VO_2_Peak. Then, the speed of the treadmill was decreased to 80% of the participant’s VO_2_Peak for the same two-minute work/recovery stages, now at 80%/50% VO_2_Peak. This new ratio was continued until the participants no longer completed an 80% two-minute stage. The treadmill speed was then lowered to 70% VO_2_Peak and finally 60% VO_2_Peak, with the additional decreasing speed cycles occurring with the same work/recovery pattern until volitional exhaustion occurred at each stage [35]. Time at each stage was recorded, as well as total EE time and distance. Participants were verbally encouraged to do their best throughout the exercise period. Water was available ad libitum throughout the EE bout and the amount (mL) consumed was recorded.

### 2.10. Rest Period Protocol

After completion of the EE, a 4 h RP began, whereby participants were allowed to rest in a semi-supine position and were only disturbed to administer feedings during the glycogen replenishment period. Throughout this period, they consumed water ad libitum. Participants completed the GSRS survey 20 min from the end of the RP.

### 2.11. Time-Trial Protocol

To help prevent pacing, all 5K time trials were completed while running on a non-motorized Curve treadmill (Woodway USA, Inc., Waukesha, WI, USA). To prevent outside influences on the performance outcome, no additional fluids or food were permitted during the trial [36]. Prior to the 5K, study participants completed a 3 min self-paced jog on the non-motorized treadmill. Then a 3 min rest commenced, followed by the 5K. All feedback on speed, time, and calories expended was removed by placing a covering over the treadmill display. Participants were able to see their distance throughout the completion of the time trial, but finish times were not revealed to them. Due to the non-blinded nature of the trial, the lead researcher did not take part in the encouragement of the participants during the time trial. Trained research team members, who were otherwise uninvolved with the trial and were uninformed as to which condition the participants’ visit entailed, provided supervision and motivation to the participants at distinct intermittent intervals throughout the 5K time trial. Verbal encouragement was given every 0.5 m extending for a tenth of a mile, and continuous from 2.75–3.1 m, using consistent motivating phrases at the different distance markings, encouraging them to finish the time trial as fast as possible. Time to completion was recorded, followed by a post-time trial blood draw within 10 min of the end of the 5K, and the recording of a post-time trial RPE immediately after the blood draw. For the RPE, the participants were asked to consider the entirety of their 5K time trial and rate their exertion level on a scale from 6 to 20, with 6 indicating the lowest level of exertion and 20 indicating the highest level of exertion.

### 2.12. GSRS Survey

The Gastrointestinal Symptom Rating Scale (GSRS) is a clinical survey developed for the analysis of GI symptoms and is derived from 15 questions consisting of a symptom paired with a scaled answer of 1–7 indicating the frequency rating of occurrence of the symptom, with one meaning ‘no symptoms’ and seven ‘severe symptoms’ [37]. The GSRS was completed upon arrival to the laboratory, immediately following the ingestion of the first serving of the conditional beverage, twenty minutes before the end of the rest period, and immediately following the post-5K RPE rating. After data collection, a mean value was calculated from the following five syndrome subcategories: diarrhea, indigestion, constipation, abdominal pain, and reflux. A GSRS total score was also calculated from the mean of the 15 questions for that participant at that timepoint and condition [37,38,39,40].

### 2.13. Blood Biomarkers

For all exercise visits, venous blood samples were collected via venipuncture of the median cubital vein upon arrival and following cessation of the 5K and analyzed for IL-6 and for I-FABP. Two SST Vacutainer™ tubes with <6 mL of blood were collected for the analyses, allowed to clot at room temperature for 30 min, and then centrifuged at 3360 rotations/min (642E-Quest, Drucker Diagnostics, Phillipsburg, PA, USA). After centrifugation, 600 μL aliquots of serum were transferred into separate labeled tubes and frozen at −80 °C for later analyses of I-FABP and IL-6. Using commercially available ELISA kits (accession numbers P12104 and P05231), the serum samples were analyzed in duplicate following manufacturer instructions (RayBiotech, Peachtree, GA, USA). Plate-to-plate controls were used between all analyzed plates with measured coefficient of variations ranging from 2.5 to 4.5% between the plates. All data were transferred to Microsoft Excel (Seattle, WA, USA) and analyzed using linear regression of unknown values against a standard curve. GraphPad Software (Insight Partners, GraphPad Holdings, v9.5.0, LLC, Boston, MA, USA) was used to generate graphical and tabular presentations of the data.

### 2.14. Adverse Event Reporting

During the duration of the study, the occurrence of adverse events was recorded through spontaneous reporting by the participants, clinical evaluation, interaction of a research team member with a study participant, or examination of a participant’s research file. The events that were recorded were systematically categorized using MedDRA system organ class and lowest level terms (LLT) before being graded using Common Terminology Criteria for Adverse Events (CTCAE), v5.0, US Dept Health & Human Services (published: 27 November 2017).

### 2.15. Statistical Analysis

All analyses were completed using Microsoft Excel and JASP (JASP Team, v. 0.18.8, 2023). Prior to performing the statistical analysis below, all variables were checked for each test’s assumptions. For all dependent measures, descriptive statistics (means and standard deviations) were calculated with the use of Microsoft Excel. Data points were considered statistically significant when the probability of type I error was 0.05 or less. The primary dependent variable was the 5K times, and the following were secondary dependent variables: IL-6, I-FABP levels, RPE, and GSRS scores. The mean differences in 5K times and the other two dependent variables, RPE and I-FABP, were assessed using a mixed factorial ANOVA with repeated measures on time to assess the time, group, and group × time interaction effects of the impact of the treatment beverage on all outcome variables. If significance was found with the repeated measures ANOVA test, then further post hoc analysis was carried out with the Holm correction factor applied. The data that were determined to be non-normally distributed were analyzed using the Friedman Test within each condition to examine changes across time, then further post hoc analysis was carried out with Conover’s post hoc tests.

## 3. Results

### 3.1. Participant Demographics

Eleven healthy men (n = 9) and women (n = 2) (55 ± 8 years, 177.3 ± 8.0 cm, 78.2 ± 14.3 kg, 24.7 ± 3.2 kg/m^2^, VO_2_Peak: 43.7 ± 6.9 mL/kg/min) (Table 1), completed all aspects of the study protocol (Figure 1). Upon arrival, compliance with the training and dietary guidelines was checked, including adherence to the high CHO diet the day before testing visits. All participants reported 100% compliance to kefir supplementation when warranted and to the pre-visit protocols; however, for dietary intake, of the 11 people who completed all aspects of the study protocol, only 10 provided suitable recorded food logs (Table 2).

### 3.2. Exhaustive-Exercise Data

The EE protocol allowed for variations in the length of time it took for participants to complete the EE each testing visit, as well as the distances they covered. Both data points were collected and analyzed with the Friedman test as neither set of data were normally distributed. No significant differences between testing visits were found for either the EE time (min) (PLA: 80.64 ± 26.86, KFR: 80.55 ± 26.63, and MLK: 80.18 ± 25.42, *p* = 1.0) or the EE length (miles) (PLA: 7.71 ± 3.85, KFR: 6.87 ± 3.72, and MLK: 7.33 ± 3.02, *p* = 0.88). The EE length between conditions was slightly variable compared to the EE time means, due to slight variations in the length at which the participants stayed at each stage of the EE.

### 3.3. Performance Metrics

The time-trial performance-time data were analyzed via a repeated measures ANOVA between the different conditions. No statistically significant group × time interaction was observed for time-trial performance times (min) (PLA: 33:39.1 ± 6:29.0 min, KFR: 33:41.1 ± 5:44.4 min, and MLK: 33:36.2 ± 6:40.5 min, *p* = 0.99). Sphericity was violated for the 5K RPE data; therefore, the Greenhouse–Geisser correction was used. No significant group x time interaction was observed for the RPE (PLA 16.78 ± 1.33, KFR: 16.72 ± 1.39, and MLK: 16.83 ± 1.74, *p* = 0.92).

### 3.4. GSRS Dimensions

The GSRS dimension data was analyzed with the Friedman test as the data was not normally distributed. No significant differences between conditions were found, but the KFR data were found to have lower means in every dimension except diarrhea (Table 3).

### 3.5. GSRS Total Scores

**Figure 4:**
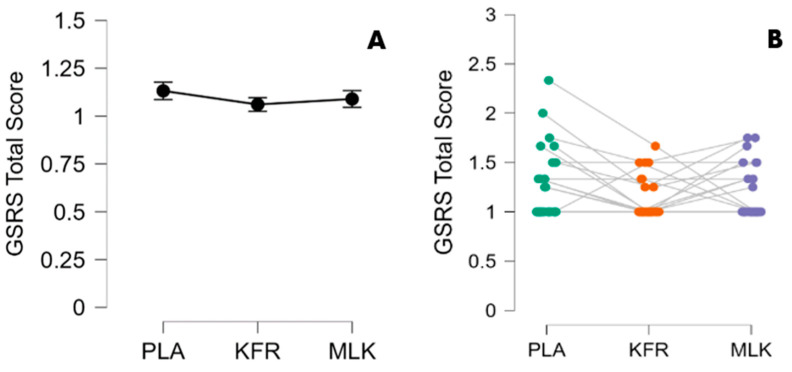
GSRS Total Scores at timepoint 2 immediately after EE for each condition (**A**) as group mean; (**B**) individual scores with each dot indicating a participant’s score when testing with each of the 3 conditions: green = PLA (placebo), orange = KFR (kefir), and purple = MLK (milk).

GSRS total score data were also not normally distributed and were therefore analyzed with the Friedman test. Significance was found at timepoint 2 (after the EE) between KFR and PLA (*p* = 0.005) with the use of Conover’s post-hoc test. Notably, KFR produced a lower (more favorable) GSRS total score compared to PLA with a 95% CI of [1.019, 1.102] and a small-to-medium effect size ($\eta_{p}^{2}$ = 0.052). No significant differences were found for KFR means at every other timepoint compared to MLK and PLA (Table 4 and Figure 4).

### 3.6. IL-6 and I-FABP Levels

All IL-6 blood levels were analyzed in duplicate and found to be below the standards used for the ELISA analysis. As a result, no data are being reported for IL-6. A total of seven participants’ I-FABP data were analyzed, as four participants’ data had to be dropped. One participant’s levels at both timepoints were found to be higher than the standards used, and two other participants had missing timepoints leading to an incomplete data set; additionally, a participant was found to have a post-time trial datapoint beyond three standard deviations of the average of their baseline levels, and was therefore assumed to be an outlier. Additionally, two participants’ I-FABP levels were above the standards and were reprocessed with a higher dilution ratio and analyzed. The data for I-FABP (n = 7) were found to be normal and were analyzed with a repeated measures ANOVA test between the three conditions at the two timepoints. No significant difference was found between I-FABP levels (ng/mL) at baseline (PLA 11.38 ± 7.48, KFR: 11.82 ± 8.95, and MLK: 10.68 ± 6.73, *p* = 0.411). After the time-trial performance bout, a significant (PLA 11.95 ± 9.63, KFR: 13.31 ± 10.83, and MLK: 11.85 ± 10.25, *p* = 0.033) (ng/mL) between-group difference was found for I-FABP levels, but follow-up Holm post-hoc testing showed no significant difference between the conditions after the time trial.

## 4. Discussion

The primary aim of this investigation was to evaluate the efficacy of KFR in promoting recovery from a challenging bout of running exercise in comparison to MLK. To our knowledge, this is the first investigation to explore the potential ability of KFR to promote recovery from challenging exercise in addition to evaluating changes in IL-6 and I-FABP and subjective ratings of GI symptoms. Our research demonstrates that the consumption of KFR or MLK did not alter performance outcomes in a 5K treadmill run, as measured against PLA. Interestingly, participants reported a general reduction in GI symptoms with the KFR post-EE, where the difference was significant compared to the PLA. It is important to note, however, that direct parallels with the existing literature are somewhat limited. This limitation stems from a scarcity of human studies focusing on the specific impact of KFR on exercise recovery, performance enhancement, or the modulation of common biomarkers.

In exploring the effects of kefir supplementation, our study enters a relatively uncharted research area. For context, Lee and colleagues observed, in a young adult cohort (20–30 y), that supplementing for 28 days with lyophilized kefir improved lactate responses and increased times to exhaustion without affecting gut microbiota [26]. Similarly, O’Brien et al. [25] noted improvements in the levels of C-reactive protein, an inflammation marker, but no enhancement in exercise performance with 15 weeks of kefir supplementation in their young adult (18–24 y) participants. To date, these are the only two controlled investigations that utilized kefir supplementation with human athletic participants in combination with some aspects of exercise performance and biomarker changes. Contrasting to these studies is an animal study by Hsu et al. [41], which reported significant improvements after kefir administration in endurance (swimming to exhaustion) and strength (forearm grip) performance in addition to improvements in biomarkers connected to metabolism and muscle damage. In addition, glycogen content was increased with kefir administration along with alterations in the gut microbiota [41]. However, drawing direct parallels between these studies and ours is complex due to the distinct differences in their designs and research questions. Our study contributes to this growing field, offering unique insights while underscoring the need for more nuanced research into kefir’s role in exercise and recovery.

Nonetheless, the glycogen replenishment protocol in this study was derived from a study performed by Dahl et al. (2019) [4], but, in regard to the PLA performance times equaling those of the KFR and MLK conditions despite the total shortage of 519 and 451.5 calories respectively, an argument can be made that the CHO recovery approaches provided to all groups during the rest period might have overpowered any differences that might have existed between the two experimental conditions. The relative dosage of 1.2 g of CHO/kg/h was used for the CHO beverage intake [1] and was given to the participants along with the granola bars during the final three hours of the recovery period. In contrast, a cycling trial in master endurance athletes by Goldstein et al. (2023) [42] compared beverages given in relative rates of CHO (1.2 g/kg) and CHO + protein (0.8 g/kg of CHO + 0.4 g/kg of protein) in a repeat exercise situation and found that both the CHO and CHO + protein conditions resulted in significantly longer test-to-exhaustion times than the placebo condition of electrolytes + water. In this study, no other supplements besides the experimental beverages were given during the 2 h recovery period that occurred between the two exercise bouts [42].

The participants in this study were asked to provide subjective ratings of their GI symptoms and functions, a crucial aspect of our protocol given the focus on probiotic supplementation. As observed in Table 3, our results for these outcomes (as measured in the GSRS) aligned with other running studies in master athletes, finding a potential benefit of the use of probiotics in athletes for improving gut health [17,22,43,44]. Notably, as detailed in Table 4, KFR supplementation, in comparison to PLA, resulted in a significantly lower total GSRS score following the exhaustive-exercise bout, illustrating the potential of KFR in mitigating GI symptoms.

**Figure 5:**
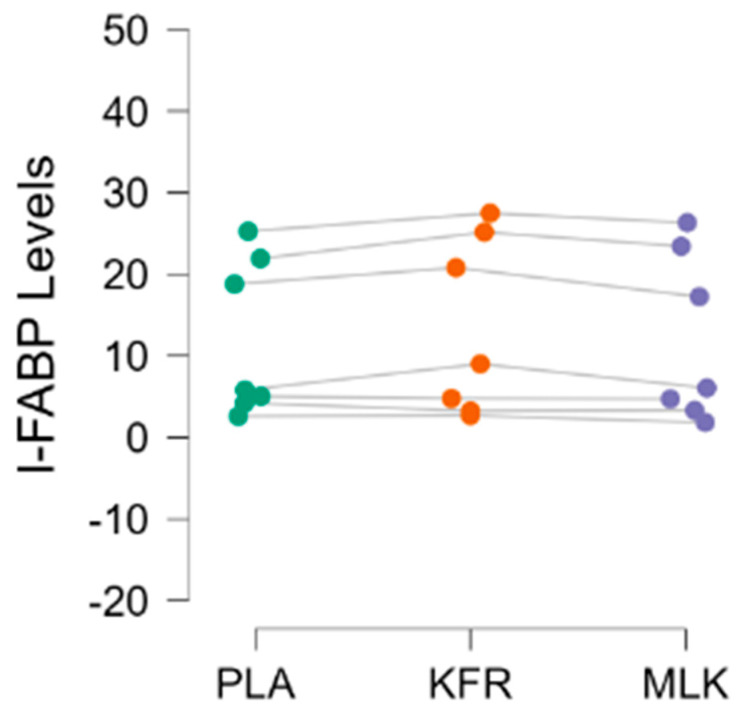
Individual I-FABP levels (ng/mL) in serum right after the time trial with each dot indicating a participant’s score when testing with each of the 3 conditions: green = PLA (placebo), orange = KFR (kefir), and purple = MLK (milk).

In addition to performance and subjective ratings, study participants provided venous blood samples before and after the completion of each study intervention to have IL-6 and I-FABP measured in these samples. Unfortunately, the determined concentrations of IL-6 in all samples were below the lowest standard and were not reported. While seemingly not related to our nutritional interventions, acute IL-6 responses in older adults have been reported to be blunted regardless of their fitness level [45], and cytokine levels in healthy (non-diseased) populations are sporadic and inconsistent [46]. Our I-FABP findings illustrate that these outcomes were quite varied, with some participants at high levels and half of our participants at low levels of our measured range (Figure 5). The I-FABP analysis revealed that the KFR group had higher I-FABP levels than the PLA or MLK groups immediately after the time trial. Higher I-FABP levels would indicate a greater level of intestinal lining damage. However, upon further Holm post hoc testing, no significance was found between the groups.

### 4.1. Strengths

A few strengths of this project should be highlighted. The first strength is the randomized, placebo-controlled, crossover approach that was employed, using a study protocol that mirrored the previous report by Karp [6]. In this respect, the crossover nature of our design, although challenging to execute, was extremely important for rigorous internal control and due to the known individualization and diversification that occurs in the gut microbiome and the associated endotoxin responses [9,47]. Our study’s focus on older endurance athletes over 39 years of age, which notably included female participants, addresses a critical gap in research, where females have traditionally been under-represented [48]. This inclusion is particularly significant given that older populations, including females, tend to exhibit higher rates of GI complications compared to their younger counterparts [15]. We implemented a high-CHO diet the day before all testing days which was set high enough to maximize the amount of stored glycogen, which eliminated any macronutrient difference in the pre-supplementation of kefir.

The selection of running as our exercise mode should be viewed as a strength due to the large external validity that exists with running and the established relationship between untoward GI responses and endurance runners [8,15,16,17]. We chose a closed endpoint exercise test (5 km time trial) due to previous research that has found these types of tests to have greater reliability when compared to open-ended tasks [36,49,50]. To minimize any learning effects, participants completed two familiarization sessions prior to beginning any nutritional interventions. A final strength was the incorporation of a food-based probiotic versus a powder or a capsulated formulation, as probiotic cultures delivered in this format commonly contain a greater number of probiotic strains and CFUs for each strain [51,52]. According to the information found on the commercial label, the KFR used in this study contained 12 live cultures and 25–30 billion CFUs at the time of manufacturing per 8-ounce serving. Additionally, of the 12 cultures contained in the kefir used, *Lactobacillus acidophilus* and *Bifidobacterium lactis* are the two most heavily researched species for the general population, while *L. rhamnosus*, *L. casei*, and *L. plantarum* have often been studied in connection with athletes [8,21,53]. The kefir for this study was consistently purchased close to the time of use for supplementation or testing to maintain the high viability of the cultures within the product.

### 4.2. Limitations

A few limitations also exist, most importantly, this trial was non-blinded. This consideration was made with much thought and analysis as we undoubtedly preferred to employ a double-blind approach, but, due to the reasons mentioned previously, there were some safety considerations that we were not able to overcome. Beyond this reason, we also accepted the very likely possibility that our participants would know what condition they were assigned due to the distinct, taste, texture, and appearance differences that exist between KFR, MLK, and PLA. To offset this known limitation, we did put several measures in place to bolster other aspects of our experimental approach, such as minimizing conversation about probiotics, fully randomizing the order in which supplements were assigned, and standardizing the completion of the 5K. To this final point, and because exercise performance was our primary outcome, for all 5K the project coordinator was not involved during the performance of the time trial as their knowledge of what condition was assigned and how much encouragement and coaching was provided could impact the final performance of each participant. Additionally, all provided feedback was standardized, with not only what was spoken (similar pattern of phrases) to the participant but also when it was spoken (every 0.5 m and the last 0.35 m). A non-motorized treadmill was used for the 5K to avoid any pacing that could occur due to the ability of a motorized treadmill to hold a constant speed. Running on a non-motorized treadmill has been shown to require both higher physiological and perceptual demand [54], but, with two familiarization trials to the time trial and the fully randomized order, it was our hope that any learning effect was negated. Another limitation relates to the supplementation protocol that was employed, as we were not able to rely on previous human studies that supplemented daily with KFR. Although adequate for the pilot nature and comparative to other trials with similar rigorous protocols [4,6,35], the small sample size of 11 that was used in this study together does limit the generalizability of our findings. The 14-day supplementation protocol employed in this study was similar in duration to two previously published studies by our research group [29,55] in addition to previously published works by Pyne et al., who highlighted that probiotic supplementation for just seven days can successfully instigate colonization in the microbiota [8]. Additionally, the three-week washout period implemented is consistent with previous research using probiotic ingestion in conjunction with crossover research designs [29,55,56,57].

### 4.3. Future Considerations

Fermented milk is considered to be one of the best vehicles for delivering probiotics as the milk proteins have the capacity to act as a buffer as it passes through the acidic stomach and the additional nutrient components from the milk provide energy for bodily functions, but also for the survivability of the microbiota [51,58,59,60]. Future studies should examine collaborations to create and deliver a KFR with the probiotics removed. In this respect, Ba et al. (2021) [61] provided yogurt smoothies with and without the addition of the probiotic *Bifidobacterium animalis* subsp. *Lactis* BB12 and also provided the probiotic strain as a capsule, and found significantly higher BB12 amounts in those that ingested the smoothie rather than in the capsulated form. If possible, this approach would facilitate effective blinding and still allow for investigations into what efficacy is afforded due to the addition of probiotics.

With respect to the currently limited amount of KFR research in athletes, many additional avenues of future research should be explored. A study that investigated the impact of long-term daily pre-exercise KFR supplementation would be beneficial to see if a longer regimen produced any effects on performance, recovery, or attenuating GI symptoms in connection with long or intense exercise bouts. Additional research can look into the underlying mechanisms that KFR supports in the reduction of gastrointestinal symptoms. Additionally, choosing additional biomarkers, such as measuring heart rate and lactate levels during the 5K, or exercise performance tests could also add more insight into any potential impact of KFR supplementation.

## 5. Conclusions

From this novel investigation, we conclude that a two-week period of supplementing with KFR prior to completing an exhaustive bout of intermittent running did not impact 5-K time-trial performance after a 4 h nutritional replenishment (recovery) period in master endurance runners. No changes were observed in the concentrations of IL-6, while the I-FABP results were largely inconclusive due to a wide range of measured values and a small set of complete data for this variable. The subjective GI symptoms reported by participants with the GSRS did identify non-significant lower dimension scores for the KFR group when compared to the PLA and MLK groups in every GI symptom category except diarrhea. Additionally, the total GSRS score for KFR was found to be statistically lower than PLA after the exhaustive-exercise bout. Due to the extremely limited number of human investigations that have explored the potential for KFR to augment human exercise recovery and performance outcomes, substantially more research is needed to fully examine KFR’s potential as a nutritional aid for exercising individuals.

## Figures and Tables

**Table 1:** Participant baseline characteristics.

	Mean ± SD	Minimum	Maximum
Age (years)	54.6 ± 7.8	40	63
Height (cm)	177.3 ± 8.0	159	190
Weight (kg)	78.2 ± 14.3	49.6	102.4
BMI (kg/m^2^)	24.7 ± 3.2	19.6	29.9
Bodyfat Percentage (%)	22.9 ± 5.6	13.6	31.9
VO_2_Peak (mL O_2_/kg/min)	43.7 ± 6.9	30.0	56.0

cm = centimeters, kg = kilograms, m = meters, mL = milliliter, O_2 =_ oxygen, min = minute, SD = standard deviation, (n = 11).

**Table 2:** Logged Dietary Intake.

	Mean ± SD	Minimum	Maximum
Average Energy for tracked days 1–2 (kcal/day)	2389 ± 524	1855	2788
Energy for High CHO day (kcal/day)	4083 ± 760	2873	5450
Average PRO for tracked days 1–2 (g/day)	107 ± 43	51	201
PRO for High CHO day (g/day)	122 ± 52	42	243
Average Fat for tracked days 1–2 (g/day)	97 ± 24	73	140
Fat for High CHO day (g/day)	123 ± 45	45	177
Average CHO for tracked days 1–2 (g/day)	280 ± 46	215	330
CHO for High CHO day (g/day)	618 ± 168	311	929
Goal CHO amount for High CHO day (g/day)	626 ± 115	397	819

Kcal = kilocalories, CHO = carbohydrates, PRO = protein, g = grams, (n = 10).

**Table 3:** Means across the timepoints for GSRS dimension scores for each condition; *p*-values with Friedman test are shown in parenthesis between conditions.

Condition	Diarrhea	Indigestion	Constipation	Ab. Pain	Reflux
PLA	1.068 ± 0.20 (0.654)	1.392 ± 0.41 (0.123)	1.045 ± 0.15 (0.603)	1.356 ± 0.55 (0.051)	1.057 ± 0.22 (0.801)
KFR	1.114 ± 0.32 (0.564)	1.375 ± 0.47 (0.084)	1.030 ± 0.16 (0.112)	1.205 ± 0.36 (0.066)	1.034 ± 0.17 (0.112)
MLK	1.076 ± 0.29 (0.121)	1.443 ± 0.51 (0.150)	1.076 ± 0.21 (0.096)	1.265 ± 0.38 (0.183)	1.068 ± 0.20 (0.392)

**Table 4:** GSRS Total Scores for each condition at each timepoint.

Condition	Baseline	Post EE	Recovery	Post 5K TT
PLA	1.197 ± 0.30	1.132 ± 0.28	1.138 ± 0.31	1.268 ± 0.53
KFR	1.223 ± 0.37	1.061 ± 0.16 *	1.168 ± 0.41	1.155 ± 0.35
MLK	1.258 ± 0.49	1.089 ± 0.21	1.200 ± 0.37	1.195 ± 0.35

Data expressed as mean ± SD (n = 11). EE = Exhaustive-exercise, TT = Time Trial; * KFR is statistically different from PLA, *p* = 0.005, with Conover post-hoc test.

## Data Availability

The datasets used and/or analyzed during the current study are available from the corresponding author upon reasonable request.
